# Venous malformation of the penis treated with a combined Nd:YAG laser and sclerotherapy technique

**DOI:** 10.1002/ski2.114

**Published:** 2022-05-27

**Authors:** Marco Fresa, Lucia Mazzolai

**Affiliations:** ^1^ Angiology Department Center for Malformation and Rare Vascular Diseases Lausanne University Hospital (CHUV) Lausanne Switzerland

## Abstract

Venous malformations are the commonest vascular anomalies and can be found everywhere in the body. Those of the external genitalia are quite rare. Although they can be small, they may give rise to psychological discomfort and functional impairment and their management can be delicate and challenging. We present a case of a large and infiltrating venous malformation of the glans penis successfully treated with a combined strategy using sclerotherapy and Nd:YAG laser.

## Graphical abstract

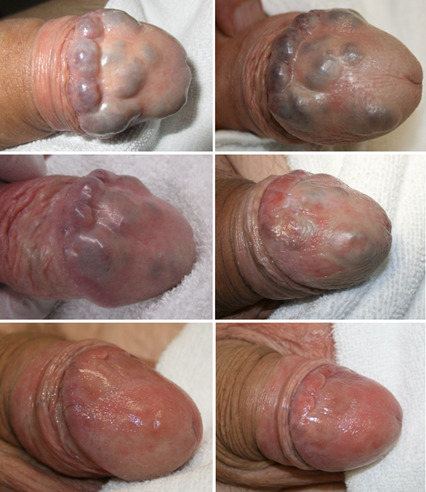

**What is already known about this topic?**

Treatment of venous malformations of the glans is still controversial. Only case reports are available concerning the treatment of these malformations.

**What does this study add?**

- Combining the available techniques could represent the key for good treatment results. We didn't find reports in the literature of clinical cases where this approach is described. This paper reports a good outcome with two techniques combined together in the treatment of a deep and extensive venous malformation of the glans penis.

## 1 INTRODUCTION

Venous malformations (VMs) are constituted by enlarged, tortuous, dysplastic vascular chambers representing the commonest congenital vascular anomalies. They can either remain undetected if deeply located until a thrombotic event occurs or they can be visible early in life when superficial or large enough to cause deformity. Venous malformations usually slowly worsen over time and never regress spontaneously. They clinically appear as blueish soft vascular masses responding to gravity as for filling and emptying their blood content. They can be found all over the body and according to their location are responsible of symptoms like pain, heaviness, swelling, ulceration, bleeding, and thrombosis.^1^, ^2^, ^3^, ^4^

VMs of the urogenital tract are very rare and those located in glans penis are the most uncommon.^2^ Case reports of penile VMs in literature are sparse.^3^ Patients are often seeking a treatment for cosmetic reason, pain relief, or functional impairment.

## 2 CASE REPORT

A 45 year old patient presented to our referral centre of malformations and rare vascular diseases for evaluation of an irregular blue‐colored mass of the glans penis. The lesion, present at birth, became significant in size at the age of 10, but at that time no treatment was offered. No other abnormality of the genitourinary tract was found in the urological follow‐up.

He denied dysuria, bleeding, hemospermia or haematuria. He did not complain of erectile dysfunction, but an important increasing in volume of the lesion during erection with evident functional impairment. We found an irregularly shaped, bluish, globular, soft vascular mass, reducible under compression, then slowly refilling in few seconds. There was no palpable thrill and the mass was not pulsatile. A colour‐Doppler ultrasound confirmed the purely venous nature of the malformation and the absence of thrombotic sequelae. Arterial and venous circulation were anatomically and functionally normal. The lesion was infiltrating the glans for 12 mm in depth, without involving the corpus spongiosum. Based on the history, physical findings and US, the diagnosis was consistent with a venous malformation, and no further imaging was performed.

A surgical option was excluded because of the wide extension of the lesion. A Neodymium:yttrium aluminium garnet (Nd:YAG) laser was proposed as a first approach. This laser, at safe energy parameters can effectively cauterise vessels at a maximal depth of 4–6 mm. Therefore, after some treatments we decided to combine the photo‐thermal ablation with sclerotherapy in order to reach the deeper parts of the lesion.

After a transdermal local anaesthesia with 5% lidocaine cream, a direct puncture of the lesion with a 23 gauges needle was performed. After slowly injecting some millilitres of 0.9% saline solution to confirm correct endoluminal positioning, we injected 1–2 ml of 3% foamed polidocanol (ratio polidocanol‐air of 1:4), under ultrasound guidance.

After removal of the needle and compressive haemostasis the lesion was further treated with application of a 1064 nm wavelength Nd:YAG laser with this parameters: 70–95 J/cm2, pulses of 30–40 msec, spots of 6–8 mm of diameter. Between 12 and 30 shots were applied per session.

All the procedures were well tolerated, without the need for further anaesthesia.

We performed in total six treatments as described above, at an interval of 6–10 weeks, with a total amount of 97 laser shots and 8 ml of total foamed polidocanol. US control was performed prior to each treatment session, demonstrating progressive fibrosis and shrinkage of the lesion.

**FIGURE 1:**
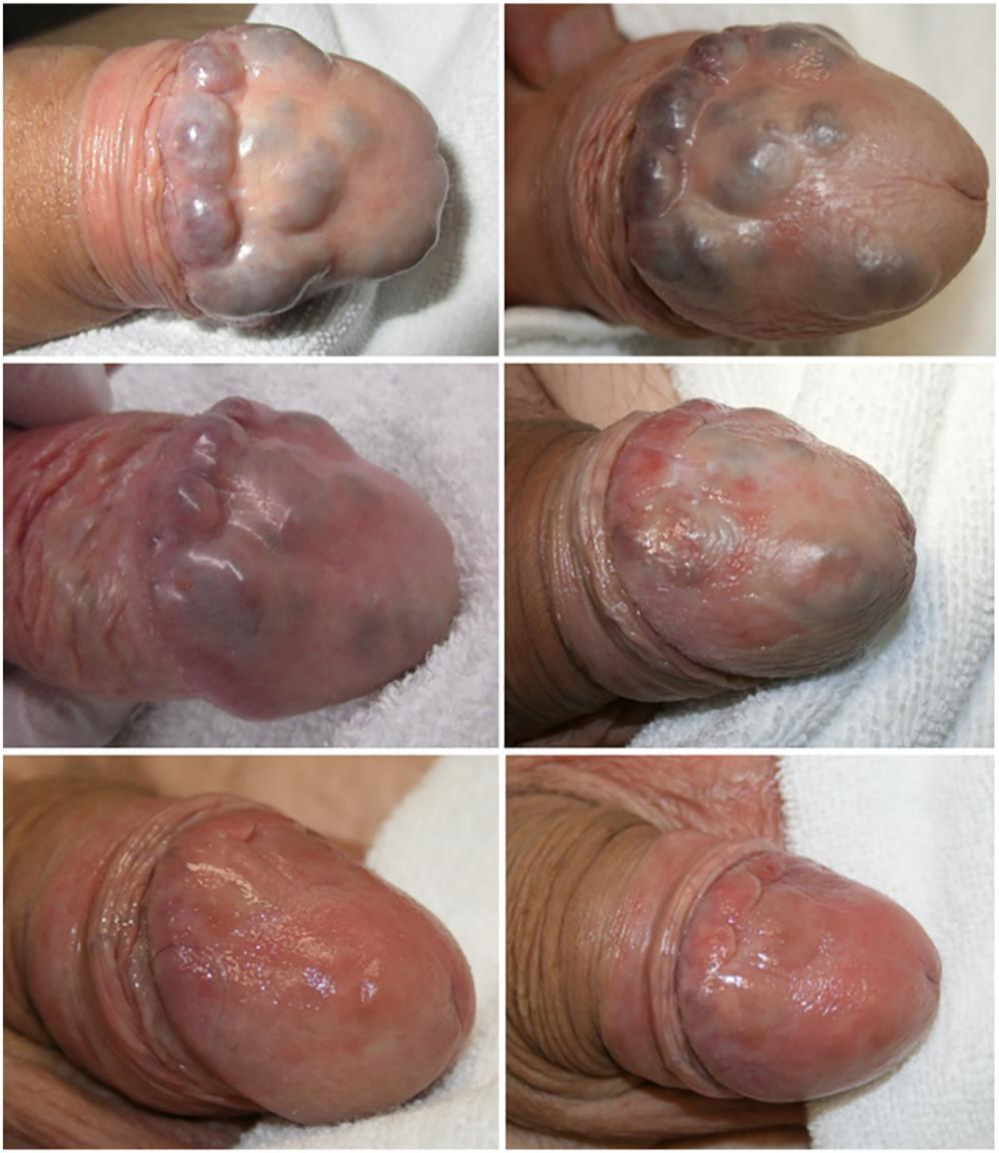
Venous malformation before and during treatment sessions. Last image correspond to 24 months follow‐up

We did not encounter any kind of complication. The outcome was very satisfying in terms of both aesthetic and functional result. US showed no thrombotic collateral damage and no residual malformation up to 24 months follow‐up (Figure 1). The patient agreed on publication of his case and imaging.

## 3 DISCUSSION

Venous malformations are congenital vascular anomalies that may slowly worsen and never regress. Those of external genitalia are quite rare. They are mostly asymptomatic, with an important aesthetic impact.^1^, ^2^, ^3^, ^4^, ^5^

A recent study showed that there could be a significant association between external and internal genital vascular malformation, thus suggesting an MRI investigation of the pelvis in all cases.^16^ For this case we didn't perform further investigations due to the advanced age of our patient, the absence of other symptoms and the fact that no other abnormality of the genitourinary tract was found in the urological follow‐up.

A review of the literature shows that current therapeutic options include surgery, sclerotherapy, cryotherapy, and laser ablation. Each technique has its indications, advantages, and possible complications.^3^, ^4^, ^5^, ^6^, ^7^, ^8^, ^9^, ^10^, ^11^, ^12^, ^13^

Surgical excision can bear good results especially for small lesions.^6^ Voluminous malformations can hardly be approached by a surgical resection, which may result in major tissue loss, and scarring could cause a urethral retraction. Moreover if a flap is necessary, the aesthetic result can be unsatisfactory.

There are no recent case reports about cryotherapy for penile lesions, probably due to technical limitations in such a small anatomic region.^15^

Nd:YAG laser treatment is better described and proved to be effective and safe; however, the available transcutaneous devices allow to only reach a depth of 4–6 mm. It should be used cautiously, to avoid mucosal necrosis.^12^, ^13^, ^14^

Sclerotherapy is a widely adopted technique for slow flow vascular malformation in every anatomical region and has proved good aesthetic and functional results even for genital lesions.^8^, ^9^, ^11^

The rationale for the association of Nd:YAG laser and sclerotherapy is to reduce as much as possible the dose of sclerosing agent and so minimise the risk of injury of the corpus cavernosum or spongiosum, that could affect the erectile function. Finally we believe that polidocanol provides a good balance between efficacy and inflammatory response, given its little impact on perivascular structures, and it is well suitable for this anatomic region, due to its low risk of ulceration and necrosis.^17^

In conclusion combined sclerotherapy and transdermal laser ablation is a promising technique for penile venous malformation. We recommend the use of cautious laser parameters and small amounts of sclerosing agent; even if the improvement after a single session may seem little, a very good final result can be obtained, without any dermal, mucosal or vascular complication.

## CONFLICT OF INTEREST

None to declare.

## AUTHOR CONTRIBUTIONS

**Marco Fresa:** Conceptualization (lead); Data curation (lead), Methodology (lead); Writing – original draft (lead); Writing – review & editing (lead). **Lucia Mazzolai:** Project administration (lead); Validation (lead); Writing – review & editing (supporting).

## Data Availability

Data sharing not applicable to this article as no datasets were generated or analysed during the current study.
